# Internet Mindfulness Meditation Intervention (IMMI) Improves Depression Symptoms in Older Adults

**DOI:** 10.3390/medicines5040119

**Published:** 2018-11-02

**Authors:** Helané Wahbeh

**Affiliations:** 1 Institute of Noetic Sciences, 101 San Antonio Rd., Petaluma, CA 94952, USA; hwahbeh@noetic.org; Tel.: +1-707-779-8230; 2 Oregon Health & Sciences University, Portland, OR 97239, USA; wahbehh@ohsu.edu

**Keywords:** older adults, depression, mindfulness meditation

## Abstract

**Background:** Older adults have fewer physiological reserves and are more likely to be affected by stress. Mindfulness meditation has the potential to be an effective treatment for depression, but little research has been conducted on older adults. The primary objective of this study was to evaluate depression symptom changes in older adults (55–80 years old) taking an Internet Mindfulness Meditation Intervention (IMMI) compared to a waitlist control. The secondary aims were to collect data on pain, perceived stress, resilience, mindfulness, sleep quality, and spirituality. **Methods:** Fifty older adults were randomized to either the Internet Mindfulness Meditation Intervention, a six-week online intervention with daily home practice, or a waitlist control. Measures were collected at baseline, after the six-week intervention period, and again six weeks later after the waitlist participants completed IMMI. Adherence to home practice was objectively measured with iMINDr. Changes in outcomes for the IMMI and waitlist participants were compared. All participants who completed IMMI were then combined for a within-participant analysis. **Results:** Adherence to the intervention was low, likely due to a traumatic event in the local area of the participants. Compared to the waitlist participants, those in IMMI had improved depression symptoms (*p* < 0.00005), perceived stress (*p* = 0.0007), insomnia symptoms ((*p* = 0.0009), and pain severity (*p* = 0.05). In the within-participant analysis of all data before and after IMMI (i.e., those initially randomized to IMMI and waitlist participants who took it), we found improvements in depression symptoms (*p* = 0.0001), perceived stress (*p* = 0.0001), insomnia symptoms (*p* < 0.00005), pain interference (*p* = 0.003), and spirituality (*p* = 0.018). A seven-week follow-up after the original six-week IMMI program showed sustained improvements in the IMMI participants. **Conclusions:** IMMI improved depression and related symptoms compared to controls despite minimal support from study staff. IMMI offers a low-dose, low-cost, easily accessible mindfulness meditation intervention for older adults with depression symptoms.

## 1. Introduction

Older adults are a quickly growing segment of the population in the United States [1]. Adults 65 years of age and older are estimated to be 20% of the population by 2050 [2]. Older adults have fewer physiological reserves and are more likely to be affected by stress and depression [3]. Late-life depression can have devastating consequences, including increased risk of morbidity and suicide, self-neglect and also lower physical, cognitive, and social functioning. These in turn can lead to higher mortality [4]. Accessible and effective interventions for elders are needed.

The low-cost, low physical and emotional risk, simplicity of administration, and self-empowerment from engaging in the practice, all support mindfulness meditation as a desirable intervention [5]. Evidence of the benefits of mindfulness meditation for many conditions is growing as a result of the exponential increase in research studies over the last 10 years. Mindfulness meditation aims to help modify individuals’ stress responses. Clinical research studies show that group mindfulness meditation is effective for stress and mood improvement [6,7] and for reducing depression symptoms in adolescents and young adults [8] as well as in adults [9]. One meta-analysis examining two common group programs, Mindfulness-Based Stress Reduction (MBSR) and Mindfulness-Based Cognitive Therapy (MBCT), administered to older adults found that MBSR reduced anxiety and depression symptoms and MBCT improved anxiety symptoms in older adults with elevated anxiety but no depression [10].

However, the in-person group format is a barrier for many older adults who would benefit from it, either because they are reluctant to participate in groups or may have limited mobility or access to group classes. Alternative mindfulness meditation delivery formats such as Internet versions are one solution and may actually be preferred to group formats. A survey on format preferences for mindfulness meditation interventions found that older adults may prefer individual or Internet interventions to groups. Of 500 survey participants, 44 were older adults between the ages of 60 and 90 years; they rated the Internet as their first-choice format (Internet 43%, Individual 39%, Group 15%) and 10% said they would refuse a group format [11].

Internet Mindfulness Meditation Intervention (IMMI) offers access to mindfulness meditation training anytime and anywhere. IMMI includes six one-hour weekly sessions with 20 min of home practice meditation between session. One study of its use in generally healthy adults reported that IMMI increased meditation practice behavior compared to a control arm that only had access to the guided meditations [12]. A pilot study in older adults found that IMMI was acceptable, feasible, and had no side effects [13].

The primary aim of this randomized controlled study was to evaluate the effects of IMMI on mood and related symptoms in older adults compared to a waitlist control. The secondary aims were to evaluate IMMI’s effects on mindfulness, pain severity and interference, resilience, and spirituality. Mood and related outcomes collected for this trial were depression symptoms, perceived stress, and sleep disturbance because of their potential sensitivity to mindfulness in this population and interaction with mood and chronic stress [14]. We hypothesized that IMMI would result in improved mood and related symptoms compared to a waitlist control.

## 2. Materials and Methods

### 2.1. Study Overview

**Figure 1:**
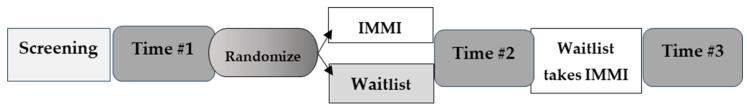
Study design. IMMI, Internet Mindfulness Meditation Intervention.

Participants underwent a screening, intervention period, and measure collection at three time points: Time #1 (before randomization), Time #2 (after IMMI, Waitlist), and Time #3 (seven weeks after Time #2). The Time #3 measurement allowed for additional data collection on IMMI effects from the waitlist participants. It also allowed for a longer follow-up from the original IMMI participants to evaluate whether any benefit of IMMI continues beyond the formal intervention period (Figure 1). During the IMMI period, participants received a one-hour session weekly for six weeks with 20–30 min of daily guided meditations for home practice. The study was approved by the Institute of Noetic Sciences Institutional Review Board Protocol Number 02-2-17-102. All subjects gave their informed consent for inclusion before they participated in the study.

### 2.2. Participants

Potential participants were screened by self-report to ensure appropriate enrollment according to the inclusion/exclusion criteria. Broad inclusion criteria were used to aid in recruitment. In order to maximize the generalizability and public health relevance of the study, exclusion criteria were minimized and based primarily on screening out participants for whom an Internet-delivered mindfulness course would not be an appropriate intervention. Inclusion criteria were: aged 55–80 years old; baseline five-item Center for Epidemiological Studies Depression Scale (CESD-5) score ≥4 [15]; stable on medications six weeks prior to and during study; willing to learn to use study technology; can hear and understand instructions; willing to accept randomization scheme and agrees to follow the study protocol; and ha own computer and Internet connection. Exclusion criteria were: cognitive impairment limiting ability to give consent or follow the protocol (≤30 on the modified Telephone Interview for Cognitive Status) [16]; significant acute medical illness that would decrease likelihood of study completion (self-report); significant, untreated depression, as assessed by CESD-5 >32 and interview; and current daily meditation practice (≥5 min/day daily for at least 30 days in the last six months. Past practice was not exclusionary but were recorded).

### 2.3. Procedures

#### 2.3.1. Recruitment

Older adults with depression symptoms were recruited primarily through: (1) Institute of Noetic Sciences (IONS) community network, (2) online listservs and research opportunities postings, (3) flyers posted at older adult community locations; and (4) outreach to older adult housing and social groups. Participants in the North San Francisco Bay Area were given preference.

#### 2.3.2. Screening

Following participant inquiries, participants were given a link to a HIPAA-compliant SurveyMonkey questionnaire, which collected self-reported information about inclusion and exclusion criteria including administering the CESD-5 [15], which ruled out untreated depression. If the participant was not eligible based on the CESD-5 score, the volunteer was given resources for mental health care. Participants who met the eligibility criteria were then invited to a telephone screening. During the telephone screening, the research assistant (RA) described the study, inclusion/exclusion criteria, risks and benefits of participation, and answered any questions by telephone. The RA conducted this screening with an approved telephone screening script to confirm eligibility. They also administered the Modified Telephone Interview for Cognitive Status (mTICS) to rule out cognitive impairment suggesting inability to give consent or do mindfulness meditation (≤30 score is exclusionary) [17].

#### 2.3.3. Time #1

Participants received an email with a link to a SurveyMonkey.com survey through which Time #1 measures were collected. Participants were then randomized to either IMMI or the waitlist control.

#### 2.3.4. Intervention Period

##### IMMI

Participants received verbal user-friendly instructions over the telephone on how to access IMMI. They also received written instructions. Participants were asked to complete their six weekly one hour sessions on their own at their convenience, although the same time and day was recommended. The RA connected with participants weekly by email to remind them of their sessions and answer any questions. This limited study staff engagement was purposeful to evaluate IMMI’s effect with minimal study support. Participants were given the RA’s phone number and email if they needed any assistance with technology or content. 

IMMI is a standardized and structured program. IMMI’s objectives are to: (1) help participants understand their personal reactions to stress, (2) teach them skills to modify their stress reactions, and (3) promote their desire for self-care and feelings of competence and mastery. Each session includes: (1) didactic instruction and discussion on stress, relaxation, meditation, and mind‒body interaction; (2) instruction and practice in formal and informal mindfulness meditation; and (3) enquiry about problem-solving techniques regarding success and difficulty in practicing mindfulness (See Wahbeh, 2012 for full curriculum) [18]. Formal meditation instruction included a mindful Body Scan and Sitting Meditation (awareness of breath, body sensations, cognitive and emotional processes). Informal practice of mindful daily activities (e.g., washing dishes) was taught to generalize mindfulness beyond the formal meditations. A 3-min meditation was also taught as a quick coping strategy.

Home practice was measured by session completion and objective iMINDr data. iMINDr is a custom software application that accurately tracks which guided meditation was listened to, how many times, and for how long they listened to it [19]. Participants were mailed an iPod Touch (Apple, Inc., Cupertino, CA, USA) with iMINDr installed and verbal and graphical instruction. Adherence was defined as session attendance (online training completion), the number of home-practice days (frequency), and the average practice time per day (duration). At the end of the six-week IMMI period, they mailed back the iPod in a self-addressed stamped envelope provided by the study staff. IMMI participants were given a link to the guided meditations online so they could continue practicing after the six-week IMMI period was over. Objective adherence was not collected during this follow-up period.

##### Waitlist

Participants in the waitlist arm received their usual and customary health and mental health care during the first six weeks of their participation. Information about any health or mental health services used during this period was tracked. After their Time #2 collection, they received IMMI as described above.

#### 2.3.5. Time #2 and #3

Measures were collected seven weeks after Time #1 and in the same manner. IMMI participants also completed a Client Satisfaction Questionnaire (CSQ), an eight-item questionnaire used to assess satisfaction with the intervention [20]. Measures were also collected seven weeks after Time #2 and in the same manner. Waitlist IMMI participants also completed the CSQ.

### 2.4. Measures (in Alphabetical Order)

Brief Multidimensional Spiritual Experiences Scale: This is a six-item scale that asks about people’s spiritual experience and is answered on a six-point Likert scale ranging from Many times a day (6) to Never or almost never (1) [21]. The score is calculated by averaging all six items. The scale has shown high reliability (α = 0.91) [22].

Brief Resilience Scale (BRS): The BRS measures a person’s perceived ability to cope with adversity. It has six items that are answered with a five-point Likert scale ranging from strongly disagree (1) to strongly agree (5). Three items are reverse scored. The item scores are averaged for the final score [23]. The BRS is reliable as a unitary construct [23] and internal consistency reliability, as measured by Cronbach’s alpha was reported 0.93 [24].

Center for Epidemiologic Studies Depression Scale (CESD): Depression was assessed during the screening procedure with a five-item subset of the original 20-item scale (CESD-5). The CESD-5 raw score was multiplied by 4 for cutoff score criteria determination. The CESD-5 has demonstrated very good sensitivity (>0.84), specificity (≥0.80), and high validity (>0.90) for in identifying patients classified as depressed by the full 20-item scale [25]. The full version was used to evaluate depression symptoms at the Time #1, #2, and #3. The CESD is a commonly used subjective measure of depressive symptoms. It asks participants about how they felt or behaved in the past week, yielding global scores ranging from 0 to 60, with higher scores indicating greater depression [26].

Five-Facet Mindfulness Questionnaire (FFMQ): Mindfulness was measured with the FFMQ, which assesses five elements of a general tendency to be mindful in daily living: observing, describing, acting with awareness, nonjudging of inner experience, and nonreactivity to inner experience [27]. The questionnaire presents a series of 39 statements and asks participants to respond according to “what is generally true for you” using a Likert scale ranging from 1 (never or very rarely true) to 5 (very often or always true). The five facets can be combined to yield a composite score that reflects a global measure of mindfulness. The global score was used in this study.

Client Satisfaction Questionnaire (CSQ-8): The eight-item Client Satisfaction Questionnaire (CSQ) [20] was administered at the measurement after receiving IMMI to assess satisfaction with the intervention. The questionnaire has demonstrated high internal consistency (α = 0.93) and strong construct validity, evidenced by correlation with service utilization and clinical outcomes [20].

Insomnia Severity Index (ISI): The ISI is a seven-item scale that asks about common sleep complaints that are answered on a 5-point Likert scale from 0–4 [28]. The score is calculated by summing all seven items and clinical categories have been designated as follows: 0–7 = No clinically significant insomnia, 8–14 = Subthreshold insomnia, 15–21 = Clinical insomnia (moderate severity), 22–28 = Clinical insomnia (severe).

Modified Brief Pain Inventory Short Form (mBPI-sf): The 11-item mBPI-sf is a two-factor questionnaire that results in scores for pain severity and pain interference [29]. Its two-factor structure has been confirmed and it has high test‒retest reliability [29]. The four pain severity items and seven pain interference items are averaged, resulting in a 0–10 range of scores for each factor.

Perceived Stress Scale (PSS): Perceived stress was measured using the PSS, a commonly used 10-item subjective instrument that measures respondents’ perceived stress in the past week [30]. It has good internal reliability (α = 0.76) and strong construct validity. The global score ranges from 0 to 36, with higher scores indicating greater perceived stress.

### 2.5. Statistical Analysis

Missing data was addressed at the participant level to minimize attrition and incomplete data. Participants were considered “dropouts” if they explicitly communicated to the study staff that they did not want to participate in the study and/or did not complete Time #2 and Time #3 surveys and did not communicate with study staff. As with most mind‒body interventions, traditional double-blinding was not feasible. Randomization was conducted with a dynamic randomization approach to help ensure that the arms were matched on age, gender, and depression score, and to reduce selection bias [31,32].

Participant characteristics at baseline were assessed with the χ^2^ test for discrete variables or analysis of variance for continuous variables. All continuous variables were evaluated with the Shapiro-Wilk test for normality. Non-normal variables were transformed as appropriate.

For analysis comparing IMMI to waitlist data (i.e., Time #1 and Time #2 measures), an analysis of covariance with Time #2 values as dependent variable, group assignment as the factor variable, and baseline Time #1 values as a continuous covariate was conducted for each measure. Pain Severity and Interference scores were square root transformed. All other variables were normally distributed. An additional analysis was conducted on Time #1 and Time #2 for IMMI participants and Time #2 and Time #3 for Waitlist participants (i.e., the period Waitlist participants received IMMI) with Student’s paired *t*-test for normally distributed variables and Wilcoxon Sign Rank test for non-normally distributed variables. False Discovery Rate correction for multiple comparisons was applied [33]. A third analysis evaluated IMMI effects over time in the IMMI group (i.e., those randomized to IMMI Time #1, Time #2, Time #3) with a repeated measures analysis of variance with each measure as the dependent variable, participant ID as a factor, and time as a repeated factor. Tukey’s honestly significant difference (HSD) test was used for post-hoc multiple comparisons between Time #1, #2, and #3. Statistical analyses used STATA 12.0 (Statacorp, LP, USA). The dataset is available on Figshare https://figshare.com/s/3d1c44e7130bb5395de0.

## 3. Results

### 3.1. Recruitment

**Figure 2:**
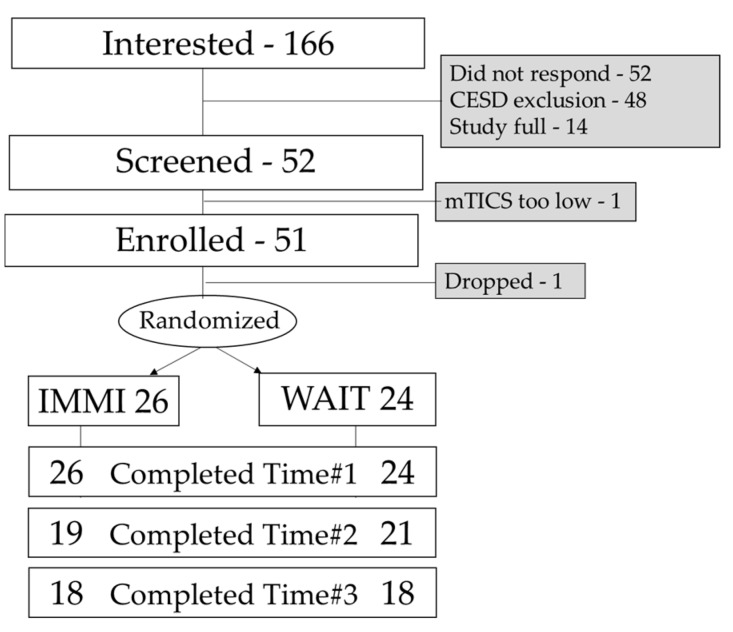
Recruitment diagram. IMMI, Internet Mindfulness Meditation Intervention; WAIT, waitlist control group; CESD, the Center for Epidemiologic Studies Depression Scale; mTICS, Modified Telephone Interview for Cognitive Status.

Study activities occurred between 16 August 2017 and 30 January 2017. One hundred sixty-six potential volunteers contacted our team with interest in the study. Forty-eight did not meet the inclusion/exclusion criteria due to the depression criteria, 52 did not respond, and 14 others contacted us after the study was already full. Fifty-two participants were screened with the mTICS and 51 met the inclusion/exclusion criteria and were enrolled and randomized. One participant dropped out before randomization. Fifty participants completed the Time #1 measures (IMMI-26, Waitlist-24); 40 completed the Time #2 measures (IMMI-19; Waitlist-21); and 36 completed the Time #3 measures (IMMI-18; Waitlist-18). Thirty-six of the 50 randomized participants completed the study for a 72% completion rate of randomized participants. See Figure 2 for the recruitment diagram.

### 3.2. Participant Characteristics

There was no difference in age, gender, education, marital status, socioeconomic status, depression symptoms, levels of stress, or cognitive function (mTICS) between the IMMI and waitlist groups (all *p* < 0.05; Table 1). Participants reported no adverse events or side effects from IMMI. Participants’ mean age was 64.8 ± 6.2 years, and they had an average of 17 ± 3 years of education. They were 88% Caucasian, 80% female, and 52% in a relationship, and there were a wide range of socioeconomic. There were more single participants in the waitlist than in IMMI.

### 3.3. Adherence

Adherence data are reported for all participants that took IMMI (i.e., IMMI and Wait_IMMI). Participants completed 3.19 ± 2.5 sessions (range 0–6), 590 ± 528 (range 0–2000) home practice minutes, and 16.4 ± 12.0 days of practice (range 0–44). A significant factor for adherence was that many of the participants were located in Sonoma County, where, during the study in October 2017, there was a large wildfire that burned 245,000 acres. Two participants were evacuated from their homes (one lost her home and pet), eight had family or friends who were evacuated, and five were physically impacted by the fires. Sixteen participants entered information in an open text field about the impact of the fires on their lives, expressing their empathy and sadness over the loss. Other participants experienced major stressors during the study period. One participant’s husband died, one participant’s mother was diagnosed with advanced cancer, one participant had surgery, and one participant’s sister-in-law died and her son’s brain cancer returned during the study independent of the fires. There were no significant correlations between session, day, or minutes practiced for any measures evaluated (all *p* > 0.05).

### 3.4. Participant Satisfaction

The participants found both interventions acceptable according to the Client Satisfaction Questionnaire. On average, participants rated IMMI above average on a scale of 1–4 (2.54 ± 0.21, range 2.1–3.1). Twenty-five participants entered text in the open field of the Client Satisfaction Questionnaire. These responses were coded as positive, negative, neutral, and positive and negative. Sixteen responses were positive, one positive and negative, three neutral, and four negative. Some example themes are represented in the following quotes.

Despite the intense stress many of the participants experienced, they reported some positive benefits to having completed the meditation program.

“I was dealing with the death of my mother just before starting this program. I realized that I was angry with her and myself. I know that the mindful practices helped me to see this. I was able to give myself and my mother’s memories more gentleness and more of an open heart and mind. Thank you!”

“I had surgery in the middle of my time. I was very fortunate that I was in the program. I did continue to keep up my meditation practice, though I got behind on the written work …. I am very glad I was chosen to participate and thank you for what you are trying to achieve. I believe the results will play a big part in the lives of older people.”

“My father died with me at his side in a local hospital two or three weeks into this program (i.e., in the middle), and my family immediately informed me that his house, where I had been living, needed to be emptied, vacated, and placed for sale within a month and a half. This set of circumstances, along with an apparently missing will, contributed to a break for my participation in this program.”

“My mom was recently diagnosed with advanced cancer, so I felt sadder than my norm the past two weeks. My answers to some questions may reflect that. Although, I believe mindfulness has helped with this as well.”

“During the time of the study my sister-in-law died and my son’s non-cancerous brain tumor has returned, so the program was especially helpful in sitting with difficult situations and compassion meditation.”

Others mentioned a shift in mental or emotional states.

“It helped me appreciate few feelings felt during meditation in the past, and look positively for meditation.”

“I have become more focused on what I am doing instead of operating on “automatic Pilot” as I did so much of the time.”

“I was so pleased with how the program had so many types of meditation to choose from and I found a couple that suited me and I am able to continue the practice on my own. The tools I learned have helped me immensely in redirecting my energies to a more positive and healthy direction. Thank you.”

“There was a noticeable decrease in depressing thoughts and stressful times.”

One specifically mentioned the benefit to their sleep patterns.

“I feel my sleep issues have greatly improved and I am very grateful to have been able to be a part of this study! Thank you!”

Others mentioned the benefit of creating a meditation practice.

“Listening to the guided meditations has become an everyday practice.”

“I’m happy to have incorporated mediation into my daily life.”

“The program met my goal of exploring again the benefits of meditative practice”.

Some participants mentioned that they did not adhere to the program as much as they would have liked to.

“It was my fault that I wasn’t able to participate as fully as I would have liked. But I did gain some tools and insights.”

“I am the worst participant ever. Such good intentions gone bad over Thanksgiving and Christmas with extensive travel and 11 grandchildren and severe flu throughout.”

There was also some feedback about improving the program. Participants who had meditation experience expressed that the curriculum was not stimulating or new to them.

“I’ve done a MBSR workshop series (in 2001), have read Full Catastrophe Living several times and used to meditate similar to this program. So, the training videos were boring to me. I was hoping for something less rudimentary but it helped me to be reminded of techniques I’ve not recalled and to have the structure to bring these back into my life. I had a friend from Holland visit week 5 and that was a big commitment that made me skip a few days of meditation. Is it necessary to have the woman in the video repeat simple info over and over? That was annoying. Then the workbook said the same thing w/o much more elaboration so I felt like it was geared to a child rather than an intelligent educated adult.”

Some participants mentioned technical issues, confusing organization, or too much information, but these comments were in a minority compared to the positive comments shown above.

“online program glitches and voice microphone issues, audio, programming glitches, some aspects of program can be improved”

“The program seems kind of jumbled up. Maybe there needs to be bullet points of all the things you are supposed to do in the week. Maybe it was just the fire which made it difficult to get all the work done. The program definitely needs some work, not too user friendly if the material is new to the participant.”

“I appreciate your efforts, it did not impress me and it was confusing at times.”

“good ideas, a few practices that have been useful, but the actual session were too cumbersome, too much narration, not a calming presentation. the amount of verbal content became work to listen to. most similar audio programs are more subtle, mellow, etc.”

Overall, the quantitative and qualitative data for participant satisfaction reflect satisfaction with the program, with a few participants commenting on improvements that could be made to the technical organization of the program.

### 3.5. Mood and Related Symptoms

Depression symptoms, perceived stress, sleep disturbance, and pain severity improved in the IMMI participants compared to the waitlist participants when examining data from Time #1 to Time #2 (Table 2).

When comparing the before and after data of all participants who completed IMMI (i.e., IMMI participants Time #1 to Time #2 and waitlist participants from Time #2 to Time #3), there were improvements in depression symptoms, perceived stress, sleep disturbances, pain interference, and spirituality (Table 3).

Examining the data for all the participants randomized to IMMI over the course of the whole study (Time #1, Time #2, and Time #3), there were sustained improvements seven weeks after the end of the IMMI program for depression symptoms, perceived stress, sleep disturbances, pain interference, and spirituality (Table 4).

## 4. Discussion

### 4.1. Summary

The current study was a pilot study examining the effects of an internet mindfulness meditation intervention on mood and related outcomes for older adults with depression symptoms. Recruitment was feasible with a 72% recruitment rate. Our randomization was effective, and the two groups were similar in important demographic and other characteristics, such as depression symptoms. Depression symptoms, sleep disturbances, perceived stress, and pain severity improved significantly more in the IMMI group than the waitlist group. Within subject analyses of all participants who took IMMI and IMMI participants over the three time points showed improvements in depression symptoms, perceived stress, sleep disturbances, pain interference, and spirituality.

### 4.2. Patient Characteristics

The two groups were similar on important demographics and depression symptoms. The study population was reflective of the locale [34]. More women participated in the study, which aligns with most complementary and alternative medicine modalities [35]. Sonoma County is also mostly Caucasian which is reflected in the distribution. The U.S. Census Bureau 2014 Annual Social and Economic Supplement mean household income was $72,641 (median $51,939) and matched our population’s income.

### 4.3. Adherence

Session adherence was lower than in other IMMI studies although home practice minutes was similar [13]. Likely, this is due to the noteworthy stressors that the group experienced, including the 2017 Northern California wildfires and other major life events. Interestingly, practice amounts were not correlated to outcomes, as has been seen in other studies [36]. Other online meditation studies have also found a high attrition rate [37]. Study staff purposefully only contacted the participants once a week with an email or voice message. Participants were given a contact number for questions but the study staff did not reach out more frequently. This was part of the study design so that we could evaluate IMMI with minimal staff support. Studies that want to maximize participant adherence should consider including additional support from the staff (e.g., scheduled weekly telephone conversations with staff). Also, the administration of iMINDr on a study iPod that is mailed to participants was not ideal and future studies should include a more feasible way of collecting objective adherence. An updated version of iMINDr has been created that can be downloaded on android or iOS devices. We do not anticipate this being a barrier for older adults as smartphone use is common even in elders. A recent study by the Pew Research Center found that 42% of U.S. adults aged 65 and older use a smartphone (that number is higher for 65–74-year-olds, 49–59%) [38].

### 4.4. Participant Satisfaction

Most of the comments in the open field of the Client Satisfaction Questionnaire were positive. These comments also highlight the increased capacity of the participants to cope with stressors in their life as a result of what they were learning in the program. There were also negative comments about technical issues, confusing format, and lack of stimulation for people who had already had meditation training. Future iterations of the program will include emphasis on reaching out for technical glitches, summary pages for each week, and also exclude participants who have had any meditation training. IMMI seems best applied as a first-exposure low-dose for mindfulness meditation. 

### 4.5. Mood and Related Outcomes

The IMMI participants had greater improvement in depression symptoms than the waitlist group in the controlled analysis. Our pilot IMMI study in this population did not see these improvements [13] nor did our IMMI study of the general population [12]. IMMI offers a low-cost, low-risk, easily accessible intervention for this high-risk population. Perceived stress also improved in the IMMI group compared to the waitlist group. These changes are reflective of other meditation studies in older adults [39] and other clinical populations [7] which observed reduction in perceived stress. Mindfulness meditation’s ability to strengthen emotion regulation [40] has been suggested as the mechanism by which participants ability to shift their perceptions of their experiences rather than rumination or focusing on the negative thoughts, and reduce experiential avoidance [41]. Sleep was also significantly improved in the IMMI group as has been noted in studies of those with insomnia and other chronic illness patients [14,42]. Pain severity also improved in the IMMI group. Pain outcomes have long been studied in mindfulness meditation intervention with increasing positive evidence [43,44,45,46] although the symptom improvements seem to mediated by psychological aspects of chronic pain [47]. These outcome improvements are remarkable considering the low-dose and high-stress that was experienced during the study period.

The improvements in the within-participant analysis including all participants who took IMMI, reflected similar outcomes as the controlled analysis with a shift from a difference in pain severity to pain interference. The pain severity outcome asks about how severe the person’s pain is, while the pain interference outcome asks about how much their pain prevents the participant from engaging in their activities of daily living. Perhaps the increased power of having additional participants in this analysis resulted in more power for a significant finding where it was not in the controlled analysis. Clearly pain outcomes are being affected but the exact mechanism in this population is not understood. Also, we were not recruiting for people with pain and so the results may imply a ceiling effect but further research on the intersection of mindfulness meditation, elders and pain are warranted. Spirituality was also improved in the within-participant analysis. Spirituality plays an important part in many people’s lives, especially elders [48]. One systematic review found that spirituality had a protective effect on cognitive decline [49] and thus, supporting elders to engage in activities that support their spirituality, whatever spirituality they choose, could support their overall health and functionality.

Because of the waitlist study design, we were able to collect a longer-term follow-up (seven weeks after the conclusion of the intervention) on the participants that were initially randomized to IMMI. Participants had access to the guided meditations after the six-week IMMI program was complete and were encouraged to continue practicing what they learned and the guided meditations. The improvements in depression, perceived stress, sleep disturbances, pain interference, and spirituality persisted after seven weeks despite numerous participants experiencing stressful events.

There are a number of limitations that should be considered when interpreting the implications of this study. Credibility and expectancy effects were not measured and should be included in future studies as they are recommended for mind‒body intervention trials in general [50]. Attrition rates were high as is often seen in low staff engagement studies. The waitlist control was chosen as an ethical control to evaluate effects [51] and to minimize disappointment in participants being randomized to a less desirable active control. Future studies would consider an active control for future studies, although ideally one that participants were excited to be in. Our previous pilot found the education active control feasible, although the participants were disappointed when they were randomized into that condition. Objective adherence was not evaluated in IMMI participants in the second phase of the study, so it is uncertain whether practice was a factor in the relatively stable improvements. Home practice was not a factor in outcomes in this study.

In conclusion, IMMI improved depression symptoms, perceived stress, and sleep disturbances with some but less evidence for improving pain severity and perhaps interference. IMMI represents a low-dose mindfulness meditation intervention that can be made available online to any older adult with depression symptoms who needs it and is unable to travel to standard group classes. IMMI and other similar online programs are an innovative option for this and other populations looking for effective therapies for their symptoms.

## Figures and Tables

**Table 1:** Participant demographic information.

Variable	IMMI (*n* = 26)	Waitlist (*n* = 24)	Statistics
Age	48.6 ± 6.1	46.9 ± 6.3	*F*(1, 49) = 0.95, *p* = 0.34
Gender (Female)	21 (81%)	19 (79%)	*X*^2^ = 0.02, *p* = 0.89
Race (Caucasian)	21 (81%)	21 (88%)	*X*^2^ = 1.92, *p* = 0.75
Relationship Couple	13 (62%)	8 (33%)	*X*^2^ = 3.98, *p* = 0.05
Single	10 (38%)	16 (67%)	
Education (years)	16.7 ± 2.9	16.7 ± 2.5	*F*(1, 49) = 0.00, *p* = 0.97
Income $0–24,999	5 (19%)	6(25%)	*X*^2^ = 5.6, *p* = 0.20
25,000–49,999	3 (12%)	6(25%)	
50,000–74,999	4 (15%)	2 (8%)	
75,000–99,999	3 (12%)	6 (25%)	
100,000 & up	11 (42%)	4 (17%)	
Depression	20.23 ± 7.8	22.5 ± 6.6	*F*(1, 49) = 1.18, *p* = 0.28
mTICS	38.2 ± 3.8	39.9 ± 3.5	*F*(1, 49) = 2.11, *p* = 0.16

**Table 2:** Outcomes of IMMI and waitlist participants.

Outcome	IMMI		Waitlist		Statistics
	Time #1	Time #2	Time #1	Time #2	
Depression	20.2 ± 7.8	15.1 ± 6.3	22.5 ± 6.6	22.5 ± 6.1	*F*(1, 39) = 13.6, *p* < 0.00005 *
Perceived Stress	19.0 ± 6.2	13.4 ± 5.2	20.4 ± 5.0	19.0 ± 5.0	*F*(1, 39) = 13.58, *p* = 0.0007 *
Sleep Disturbance	13.3 ± 6.3	9.3 ± 6.0	12.3 ± 4.5	12.9 ± 4.4	*F*(1, 39) = 13.15, *p* = 0.0009 *
Mindfulness	3.1 ± 0.4	3.0 ± 0.4	3.0 ± 0.3	3.1 ± 0.3	*F*(1, 39) = 1.19, *p* = 0.28
Pain Interference	2.5 ± 2.6	1.4 ± 1.7	2.1 ± 1.7	1.5 ± 1.5	*F*(1, 39) = 2.49, *p* = 0.12
Pain Severity	2.3 ± 1.9	1.2 ± 1.7	1.7 ± 1.3	1.8 ± 1.4	*F*(1, 39) = 4.31, *p* = 0.05 *
Resilience	2.0 ± 0.3	2.1 ± 0.3	2.1 ± 0.2	2.1 ± 0.3	*F*(1, 39) = 0.55, *p* = 0.46
Spirituality	23.0 ± 7.5	24.6 ± 8.2	19.1 ± 6.8	19.8 ± 7.5	*F*(1, 39) = 1.87, *p* = 0.18

Note: Pain severity and interference were not normally distributed and were square root transformed prior to ANOVA analysis. * Significant using false discovery rate correction for multiple comparisons.

**Table 3:** Outcomes before and after IMMI for all participants (*n* = 39).

Outcome	Before	After	Statistics
Depression	22.8 ± 5	18.7 ± 6.6	*t*(37) = 4.6, *p* = 0.0001 *
Perceived Stress	19 ± 5.7	14.1 ± 6	*t*(37) = 4.27, *p* = 0.0001 *
Sleep Disturbance	13.1 ± 5.5	8.3 ± 5.8	*t*(37) = 5.63, *p* < 0.00005 *
Mindfulness	3.1 ± 0.3	3.0 ± 0.3	*z*(37) = 1.52, *p* = 0.13
Pain Interference	2.1 ± 2.2	1.4 ± 1.8	*z*(37) = 2.95, *p* = 0.003 *
Pain Severity	2.1 ± 1.7	1.5 ± 1.7	*z*(37) = 1.74, *p* = 0.08
Resilience	2.0 ± 0.3	2.1 ± 0.2	*z*(37) = −1.12, *p* = 0.27
Spirituality	21.6 ± 7.6	23.4 ± 8.4	*t*(37) = −2.47, *p* = 0.018 *

* Significant using false discovery rate correction for multiple comparisons.

**Table 4:** Outcomes for IMMI only participants Time #1, Time #2, Time #3.

Outcome	Time #1 (*n* = 26)	Time #2 (*n* = 19)	Time #3 (*n* = 18)	Statistics
**Depression**	22.5 ^a^ ± 5.7 ^a^	18.5 ^a^ ± 5.2	18.6 ± 8.9 ^a^	*F*(2, 62) = 5.95, *p* < 0.00005 *
**Perceived Stress**	19.0 ^d^ ± 6.2 ^d^	13.4 ^d^ ± 5.2	11.9 ± 6.1 ^d^	*F*(2, 62) = 23.3, *p* < 0.00005 *
**Sleep Disturbance**	13.3 ^b^ ± 6.3 ^b^	9.3 ^b^ ± 6.0	9.3 ± 6.3 ^b^	*F*(2, 62) = 9.22, *p* = 0.0006 *
**Mindfulness**	3.1 ± 0.4	3.0 ± 0.4	3.0 ± 0.4	*F*(2, 62) = 0.88, *p* = 0.42
**Pain Interference**	2.5 ^b^ ± 2.6	1.4 ^b^ ± 1.7	1.8 ± 2.5	*F*(2, 62) = 4.83, *p* = 0.01 *
**Pain Severity**	2.3 ± 1.9	1.2 ± 1.7	1.8 ± 2.7	*F*(2, 62) = 2.63, *p* = 0.09
**Resilience**	2.0 ± 0.3	2.1 ± 0.3	1.9 ± 0.3	*F*(2, 62) = 3.05, *p* = 0.06
**Spirituality**	23.0 ^b^ ± 7.5	24.6 ± 8.2	25.8 ^b^ ± 7.4	*F*(2, 62) = 5.29, *p* = 0.0098 *

Notes: * Significant using false discovery rate correction for multiple comparisons. Matched numbers represent significant paired comparisons at the following *p*-values ^a^ *p* < 0.05; ^b^ *p* < 0.01; ^c^ *p* < 0.001; ^d^ *p* < 0.00005.
